# Tunable Diode Laser Absorption Spectroscopy Based Temperature Measurement with a Single Diode Laser Near 1.4 μm

**DOI:** 10.3390/s22166095

**Published:** 2022-08-15

**Authors:** Xiaonan Liu, Yufei Ma

**Affiliations:** National Key Laboratory of Science and Technology on Tunable Laser, Harbin Institute of Technology, Harbin 150001, China

**Keywords:** tunable diode laser absorption spectroscopy (TDLAS), double-line thermometry, temperature measurement, McKenna burner, scramjet model engine

## Abstract

The rapidly changing and wide dynamic range of combustion temperature in scramjet engines presents a major challenge to existing test techniques. Tunable diode laser absorption spectroscopy (TDLAS) based temperature measurement has the advantages of high sensitivity, fast response, and compact structure. In this invited paper, a temperature measurement method based on the TDLAS technique with a single diode laser was demonstrated. A continuous-wave (CW), distributed feedback (DFB) diode laser with an emission wavelength near 1.4 μm was used for temperature measurement, which could cover two water vapor (H_2_O) absorption lines located at 7153.749 cm^−1^ and 7154.354 cm^−1^ simultaneously. The output wavelength of the diode laser was calibrated according to the two absorption peaks in the time domain. Using this strategy, the TDLAS system has the advantageous of immunization to laser wavelength shift, simple system structure, reduced cost, and increased system robustness. The line intensity of the two target absorption lines under room temperature was about one-thousandth of that under high temperature, which avoided the measuring error caused by H_2_O in the environment. The system was tested on a McKenna flat flame burner and a scramjet model engine, respectively. It was found that, compared to the results measured by CARS technique and theoretical calculation, this TDLAS system had less than 4% temperature error when the McKenna flat flame burner was used. When a scramjet model engine was adopted, the measured results showed that such TDLAS system had an excellent dynamic range and fast response. The TDLAS system reported here could be used in real engine in the future.

## 1. Introduction

Fast and stable measurement of the temperature and the concentration of combustion products in the combustion field is crucial for improving the combustion efficiency of the engine, optimizing the combustion control strategy, and reducing pollutant emissions [1,2,3]. At the same time, the complexity of the combustion process in engines, which involves high temperature, high pressures, and strong turbulence, poses a high challenge for measurement techniques [4]. However, traditional contact temperature measurement methods have the disadvantages of long response time, single-point measurement, and different degrees of interference to the combustion field [5,6], which cannot meet the existing testing requirements. Therefore, a non-contact combustion field temperature and gas concentration detection method with fast and high accuracy has an important value in the combustion diagnosis of engines.

The non-contact method analyzes the temperature change trend in the combustion field through the response of acoustic [7] or optical [8] signals. However, the acoustic methods are easily affected by the complex test environment and products within the combustion field. With the merits of high sensitivity, online measurement, and fast response [9,10,11,12,13,14,15,16,17,18,19,20,21,22,23], optical methods are based on various interaction modes of light and matter to obtain the temperature and concentration, including coherent anti-Stokes Raman spectroscopy (CARS) [24] and planar laser-induced fluorescence (PLIF) [25]. At present, these studies have been preliminarily applied to flame temperature and plenty of optics elements, and a clean operating environment, which are often difficult to meet at the test site.

The rapid development of near-infrared narrow linewidth diode lasers in recent years has facilitated the applications of tunable diode laser absorption spectroscopy (TDLAS) in flame measurement [26,27,28,29,30]. The TDLAS technique was widely used in combustion field diagnosis due to its advantages of high sensitivity, strong stability, and fast response [31,32,33]. The double-line temperature measurement method in TDLAS can effectively obtain the temperature of flame by detecting the changes in the two characteristic spectral absorption lines. For example, Farooq, A. et al. used the carbon dioxide (CO_2_) absorption lines at 3633.08 cm^−1^ and 3645.56 cm^−1^ to achieve the detection of flame temperature in a shock tube [34]. However, water vapor (H_2_O) has a wider spectral line coverage, and its content is higher than other products in the combustion process, so its absorption lines are more suitable for temperature measurement. Chang Liu [35] and Li Fei [36] et al. selected the absorption lines of H_2_O at 7185.597 cm^−1^ and 7444.350 + 7444.370 cm^−1^ (combined) and carried out temperature tests for flat flame burner and scramjet, respectively. Qu Zhechao et al. [37] proposed an in-reactor H_2_O temperature test based on calibration-free wavelength modulation spectroscopy (CF-WMS) using the 7153.74 cm^−1^ and (7154.35 + 7154.59 cm^−1^) combined absorption lines. Furthermore, some other similar works of TDLAS-based temperature measurement were also reported [38,39,40]. However, due to the limited tuning range of the diode laser, two diode lasers are usually required to cover two spectral lines. In addition, wavenumber detection devices such as etalons are added to the system to avoid errors caused by laser wavenumber shifts. These problems greatly increase the complexity and instability of the processing of the test system.

In this invited manuscript, a diode laser with a center wavelength of 1397.8 nm capable of simultaneously covering two H_2_O absorption lines was used for the temperature measurement in the TDLAS system based on the direct absorption method. The use of a single laser has the advantages of a simple system structure, reduced cost, and increased system robustness. Furthermore, the spectral line intensity of the absorption lines with wavenumbers of 7153.749 cm^−1^ and 7154.354 cm^−1^ was as low as 10^−7^ cm^−2^atm^−1^ under room temperature, which is about one ten thousandths of the spectral line intensity in the high-temperature region. Therefore, when the optical path length in the air is less than 0.1 times the effective absorption range, the absorption of H_2_O in the test environment during the measurement can be neglected. In addition, two absorption lines covered by one diode laser were used to calibrate the laser wavelength, which overcomes the error caused by the wavelength shift of the laser and requires no additional wavelength detection device. Experimental measurements were carried out for the flat flame burner and the scramjet model engine, respectively. The TDLAS system showed excellent temperature measurement performance. The system is expected to be applied in the combustion field diagnosis of real engines in the future.

## 2. Selection of H_2_O Absorption Lines

The appropriate selection of spectral absorption lines is essential for temperature measurement in TDLAS [41]. H_2_O is one of the main products of alkane fuel combustion and contains a large number of spectral absorption lines in the near-infrared band. Compared to lasers and detectors in the mid-infrared band, diode lasers and detectors in the near-infrared band are mature and inexpensive and do not require operation at low temperatures [42,43], which is beneficial to practical applications. In addition, lasers in the optical fiber communication band (1.25 µm to 1.65 µm) can be transmitted over long distances. Selecting absorption lines in this band can keep the instrument away from the test site, to avoid the impact of the on-site interference on the TDLAS system. Based on the above analysis, H_2_O spectral absorption lines in the optical fiber communication band will be selected in this research.

According to the HITRAN 2012 database [44,45], a large number of H_2_O absorption lines exist near 1.3 µm in the overtone region. Many of them have a spectral line intensity of ~10^−4^ cm^−2^atm^−1^. The selection principle of absorption lines can be considered from the following aspects. Firstly, the selected absorption lines cannot be interfered with by other absorption lines. Secondly, the absorption coefficient of the selected lines should meet the test requirements. When the effective optical path length was determined, the absorption rate mainly depends on the absorption coefficient. Therefore, to obtain a high signal-to-noise (SNR) ratio, when the optical path length is less than 10 cm, the absorption coefficient should not be lower than 10^−4^ cm^−1^. Finally, to ensure the high sensitivity of the system for temperature measurement, the energy difference between the low transition states of the two selected spectral lines should not be lower than 700 cm^−1^.

**Figure 1:**
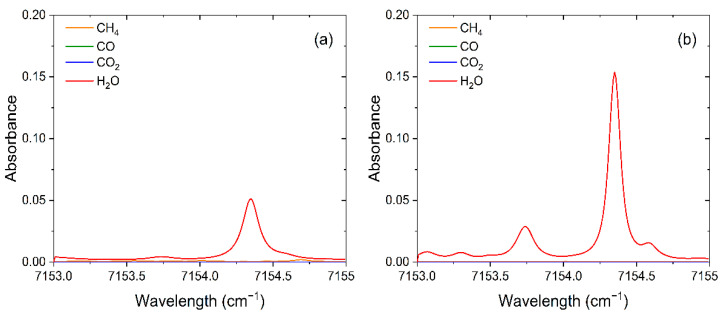

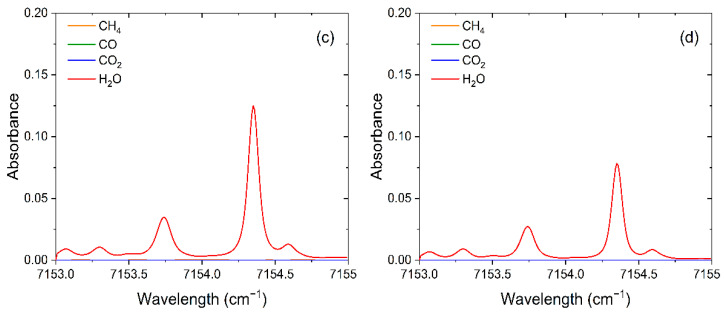
The absorption lines of CH_4_, CO, CO_2,_ and H_2_O in the range of 7153–7155 cm^−1^ at different temperatures (P = 1 atm, L = 10 cm). (**a**) 500 K. (**b**) 1000 K. (**c**) 1500 K. (**d**) 2000 K.

In this experiment, two H_2_O absorption lines located at 7153.749 cm^−1^ (L1) and 7154.354 cm^−1^ (L2) were selected as the target lines for temperature measurement using double-line thermometry. The absorbance of H_2_O, methane (CH_4_), carbon monoxide (CO), and CO_2_ which may be present in the test environment at different temperatures was analyzed at concentrations of 30%, 30%, 5%, and 30%, respectively. It can be found from Figure 1 that CH_4_ and its combustion products of CO and CO_2_ do not interfere with the target absorption lines. Furthermore, wide fluctuations in temperature can cause changes in spectral line broadening, so the effect of temperature on spectral linewidth must be considered. If the absorption lines are too close to each other, it will lead to undesirable test results. The trends of the absorbance of the target absorption lines at different temperatures in Figure 1 were obtained from simulations based on the Voigt line function. It can be found that the target absorption lines in the range of 500 to 2500 K do not interfere with each other, which proves the applicability of the target absorption lines for temperature measurements.

According to the principle of double-line pyrometry, the ratio of the intensity of the two absorption lines is the same as the ratio of the spectral absorbance integral value, as shown in Equation (1):

(1) $${\left(\frac{A_{1}}{A_{2}}\right)}_{T}={\left(\frac{-ln{\left(\frac{I_{t}}{I_{0}}\right)}_{1}}{-ln{\left(\frac{I_{t}}{I_{0}}\right)}_{2}}\right)}_{T}={\left(\frac{{\left(SPLX\right)}_{1}}{{\left(SPLX\right)}_{2}}\right)}_{T}={\left(\frac{S_{1}}{S_{2}}\right)}_{T}=R$$

where *A_i_* is the integral value of the spectral absorbance corresponding to the different absorption lines (*i* = 1, 2); *I*_0_ and *I_t_* are the light intensities before and after crossing the area to be measured, respectively; *P* is the pressure [atm]; *T* is the temperature [K]; *L* is the effective light path length [cm]; *X* is the gas concentration; *S_i_* is the spectral intensity of the different absorption lines (*i* = 1, 2) [cm^−2^atm^−1^]; *R* is the absorption line intensity ratio. In addition, the measurement sensitivity of temperature can usually be expressed as the differentiation of the ratio of spectral line intensity *R* against the temperature *T*, as shown in Equation (2):

(2) $$\left|\frac{\mathrm{dR}/R}{\mathrm{dT}/T}\right|=\left(\frac{\mathrm{hc}}{k}\right)\frac{\left|\Delta E^{\prime \prime }\right|}{T}$$

where *h*, *c*, and *k* are Planck constant [J·s], lightspeed [m/s], and Boltzmann constant [J/K], respectively; Δ*E*″ is the low state energy level difference [cm^−^^1^].

The main parameters of wavenumber (*v*), line strength (*S*), lower state energy (*E*″), air-broadened half-width (*γ_air_*), and self-broadened half-width (*γ_self_*) for the target absorption lines are listed in Table 1. The wavenumber difference between the target absorption line pairs is greater than 0.605 cm^−1^, which can be covered simultaneously in a single scan period by a continuous wave distributed feedback (CW-DFB) diode laser. Therefore, the laser wavenumber can be calibrated using the absorption peak position. The energy level difference between the two absorption lines is Δ*E*″ = *E*_1_ − *E*_2_ > 700 cm^−1^, which can meet the test requirements. At room temperature and standard pressure, the absorption coefficient of the target absorption lines is about 10^−7^ cm^−1^, which is much lower than 10^−4^ cm^−1^ in the test region. Therefore, when the absorption coefficient in the air is much smaller than the region of interest, the interference caused by H_2_O in the environment can be ignored, which can ensure a high test accuracy of the system.

**Figure 2:**
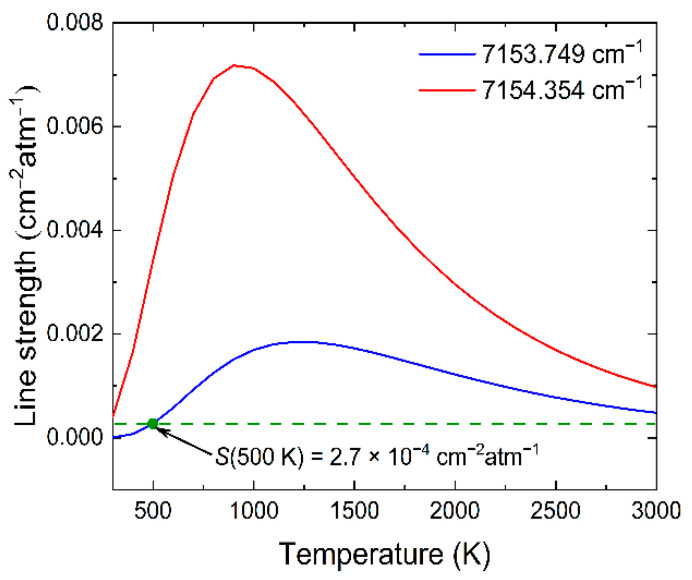
Line strength as a function of temperature.

**Figure 3:**
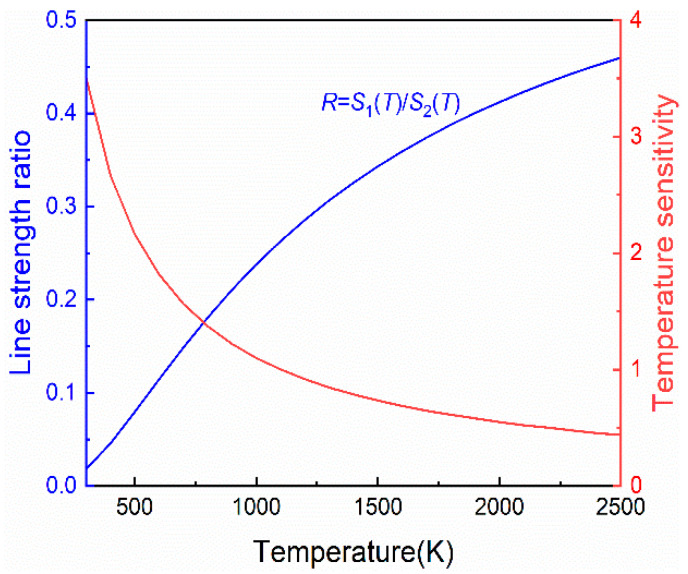
Line strength radio and sensitivity as a function of temperature.

Figure 2 shows the variation trend of the spectral intensity of the two H_2_O absorption lines with temperature. In the temperature range of T < 400 K, the intensity of selected absorption lines is much lower than 10^−4^ cm^−2^atm^−1^, so the H_2_O absorption in the environment can be neglected. When the temperature is greater than 500 K, the spectral line intensity S(T) > 2.7 × 10^−4^ cm^−2^atm^−1^, which can ensure that the measured signal has a high SNR and meet the temperature measurement requirements. According to the sensitivity calculation Equation (2), it can be known that the greater the energy level difference Δ*E*″ produces the higher the temperature sensitivity. As shown in Figure 3, the sensitivity value of this TDLAS system is greater than 0.47 in the entire temperature range (500 to 2500 K), which ensures high sensitivity for the test system to temperature measurement.

## 3. Experimental Setup

**Figure 4:**
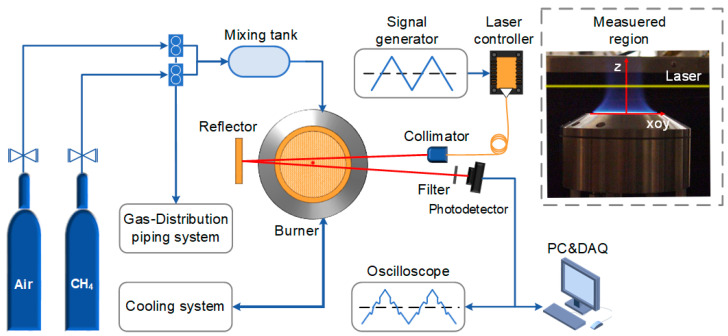
Schematic diagram of TDLAS sensing system.

The schematic of the reported TDLAS system is depicted in Figure 4. A CW-DFB diode laser (NEL NLK1E5GAAA) emission at 1397.80 nm was chosen as the excitation source. The laser was driven by a laser controller (Healthy Photon DFB-2000) whose drive signal was derived from a triangular wave generated by a signal generator. The laser beam was collimated and sent to the burning area of the flat flame (McKenna standard burner). The collimator (*f* = 30 mm) was fixed on the *z*-axis stage by a clamp, and fine adjustment down to ~10 µm can be achieved by an adjustable platform. A gold-coated reflector (Thorlabs CM508) was used to refract the laser beam back to a photodetector for twice absorption. The signal from the photodetector (Thorlabs DET20C/M) was finally collected by a data acquisition card (DAQ, Healthy Photon USB2066) and uploaded to the computer. A narrow-band filter (Thorlabs FB1400-12) was placed in front of the photodetector to reduce disturbances such as the spontaneous emission spectrum generated during the combustion process. The laser beam was adjusted to pass through the center of the flat flame burner as it travels across the combustion area to ensure an adequate absorption path.

The McKenna burner [46,47,48] was chosen in the experiment to generate a flat flame for the test. The certified gas CH_4_ and air were used as the fuel and accelerant for the flat flame burner, respectively. The flow rates of CH_4_ and air were controlled separately by two mass flow meters to achieve the change of the burner equivalence ratio. The mass flow meter has a systematic error of about 3%, which affects the accuracy of the equivalence ratio setting slightly. The flat flame burner is capable of producing a disc-shaped flame [40] with a burning area of approximately 60 mm in diameter. The Cartesian coordinate system was constructed with the center of the flat flame burner as the coordinate origin. The direction along which the flame burns was defined as the z-direction.

**Figure 5:**
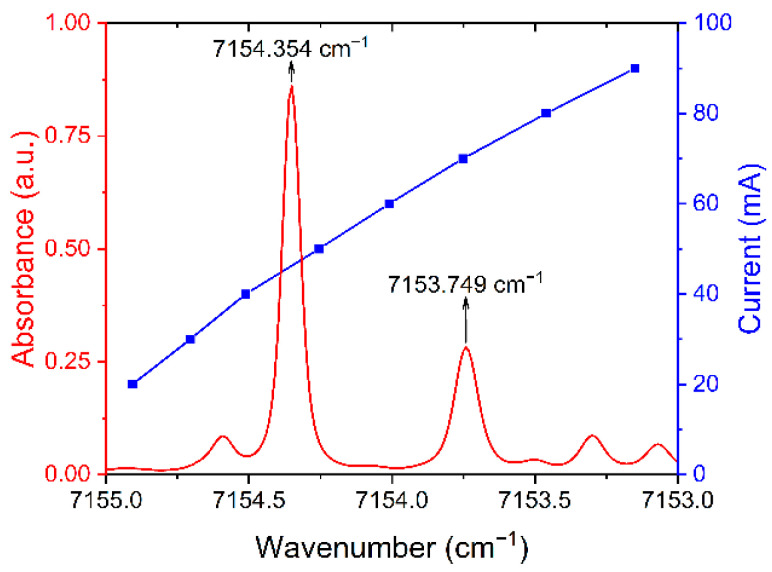
The scanning range of the used CW-DFB diode laser.

The output wavenumber of the diode laser can be controlled using temperature tuning and current tuning. In this experiment, the CW-DFB laser can cover the two selected absorption lines simultaneously in a single scanning period, and its scanning range was shown in Figure 5. The wavelength tuning coefficient of the current was 0.023 cm^−1^/mA. In the investigations, the laser temperature was set to 18.5 °C, and the current range of the sawtooth wave was from 25 to 90 mA. The two absorption peaks correspond to currents and powers of 49 mA, 72 mA, 12.55 mW, and 18.16 mW, respectively. However, the long-term operation of the laser and environmental changes can cause a wavenumber shift, which affects the accuracy of the TDLAS system. Usually, an additional device of etalon [49], wavelength meter [50], etc., are used to monitor the changes in laser wavenumbers. In this manuscript, the relative positions of target absorption lines were used to determine the interval of scanning wavenumbers. Therefore, the wavelength tuning coefficient for the scanning current can be corrected timely. According to this coefficient, the trend of the absorption ratio as a function of time can be obtained. The system sampling rate determines the resolution of wavenumber calculation, and the resolution of wavenumber increases with the increase in the sampling rate. In this experiment, the sampling rate of the system was set to 1 MSa/s, which is about 1000 times that of the triangular wave signal.

## 4. Results and Discussion

The temperature of the flat flame burner at the equivalence ratios (*φ*) of 1.0, 1.1, 1.2, 1.3, and 1.4 was measured by this TDLAS method with a scanning rate of 1 kHz. The CH_4_ flow rate was set to 1.733 L/min, and the airflow rate was set to 16.50 L/min, 14.96 L/min, 13.70 L/min, 15.00 L/min, and 11.80 L/min, respectively.

**Figure 6:**
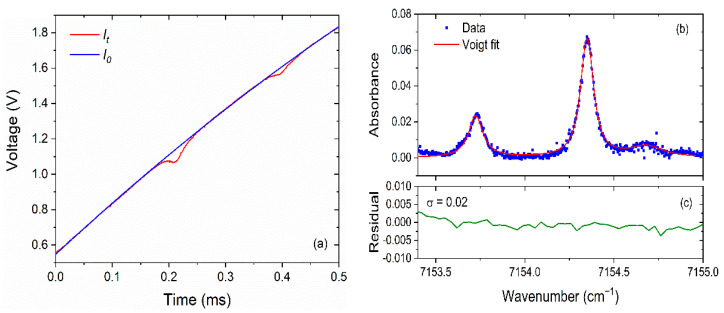
The measured signal at an equivalence ratio *φ* of 1.0 and laser height of 15 mm. (**a**) Light intensity signal captured by photodetector. (**b**) The absorbance of the two H_2_O absorption lines. (**c**) The residual of experimental data and double-line Voigt fit data.

Figure 6 shows the measured signal using the TDLAS system when the equivalence ratio of CH_4_ was 1.0 and the height (*z*) was 15 mm. The transmitted light intensity *I_t_* in the time domain was captured by the photodetector after the laser beam passes through the burning region. The initial light intensity *I*_0_ was obtained by baseline fitting with It. According to the positions of the absorption peaks of H_2_O, the wavenumber calibration of the light intensity signals can be carried out. Based on Beer–Lambert law the absorbance of the two lines was obtained as a function of wavenumber. Using the Voigt multi-peak fitting function, the absorbance was fitted, and the results were shown in Figure 6b. Comparing the fitted absorbance curve with the test data, the residual was shown in Figure 6c, and the standard deviation was 0.02. The integrated area *A* corresponding to the absorption peak was obtained, respectively, and the corresponding temperature values can then be derived using the principle of double-line thermometry [26,27,28].

**Figure 7:**
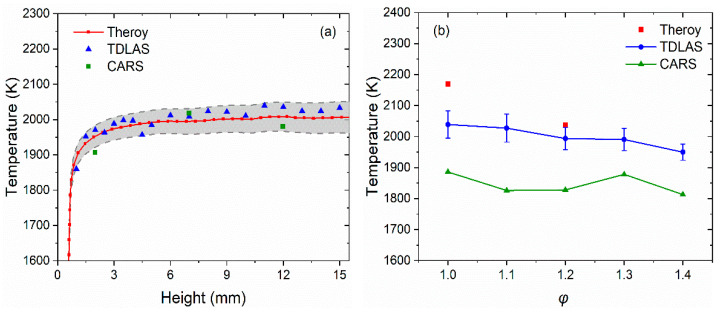
Comparison of temperature for a McKenna flat flame burner using different methods. (**a**) The flame temperature at different heights with the *φ* of 1.0. (**b**) The temperature of the flame at different *φ* at a height of 15 mm.

By gradually changing the height of the laser beam across the burning region in the *z*-axis direction, the flame temperatures were measured as shown in Figure 7a. It can be found that the flame temperature firstly increased with increasing height and then gradually stabilized at ~2000 K. Compared with the values obtained using the CARS method and theoretical calculation [51], it was found that the relative errors of the TDLAS results were less than 4% and were all distributed within the error band. Subsequently, the laser height was fixed at 15 mm to measure the flame temperature distribution at different equivalent ratios. The obtained results in Figure 7b showed that the flame temperature decreases as the equivalent ratio increases. CH_4_ fuel in the rich combustion state did not burn sufficiently, so less heat was released, and the temperature decreased. The temperature measured by TDLAS for different combustion equivalent ratios followed the same trend as the temperature measured by CARS technique and theoretical calculation [47]. The error bar was as small as 70 K at the same equivalent ratio, which may be caused by the flame jitter in the combustion process.

**Figure 8:**
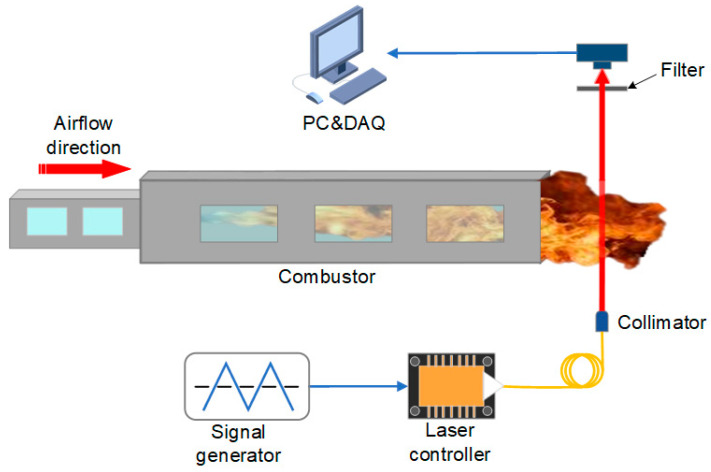
Schematic diagram of the TDLAS system for scramjet model engine.

**Figure 9:**
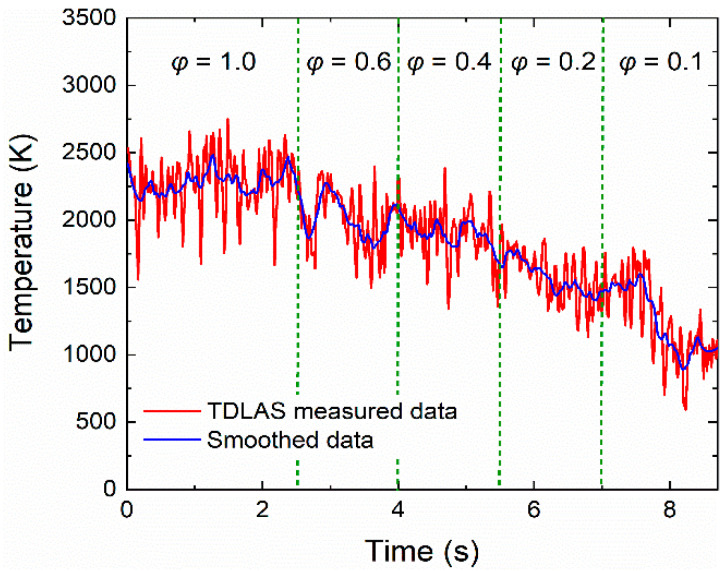
Dynamic temperatures of the tail flame for a scramjet model engine.

To further verify the performance of such TDLAS technique, temperature measurement was performed on a scramjet model engine. The schematic diagram is shown in Figure 8, with the laser beam passing through the tail flame region directly. Kerosene was used as the fuel for the scramjet model engine, and the combustion products included H_2_O, CO, and CO_2_. The combustion process in a scramjet model engine is much more violent than in a flat flame burner. Therefore, to guarantee the test stability, the reflector was removed, and the frequency of the triangular wave was increased to 3 kHz. Figure 9 shows the trend of temperature variation in the tail flame region of the scramjet model engine. With the decrease in fuel equivalence ratio, the tail flame temperature gradually decreased from 2250 K to 900 K. The TDLAS system was able to respond quickly to temperature changes by adjusting the fuel equivalent ratio in 1.5 s intervals. Compared to the McKenna flat flame burner, the temperature testing results from the scramjet model engine have suffered high jitter noise, which can be attributed to the strong turbulent flow field in the tail flame region. This TDLAS system had shown an excellent response capability and a wide temperature measuring range during the measurement of the scramjet model engine.

## 5. Conclusions

A TDLAS system based on a single diode laser was developed for temperature measurement with a large dynamic range and high measurement accuracy. A CW-DFB diode laser with an emission wavelength of near 1.4 μm was selected to cover the two H_2_O absorption lines located at 7153.749 cm^−1^ and 7154.354 cm^−1^, simultaneously. The use of a single laser has the merits of a simple system structure, reduced cost, and increased system robustness. In addition, two absorption lines covered by one diode laser can be used to calibrate the laser wavelength, which overcomes the error caused by the wavelength shift of the diode laser and requires no additional wavelength measurement device. The line intensity of these two absorption lines under standard conditions (room temperature and 1 atm) with 10^−7^ cm^−2^atm^−1^ is about one ten thousandths of the spectral line intensity in the high-temperature region (500 to 2500 K). If the optical path length in air is less than 0.1 times the interesting region, the measurement error in the TDLAS system caused by environmental H_2_O is avoided. The wavenumber interval of the target absorption line pairs was 0.605 cm^−1^ and the low-state transition energy level difference was greater than 700 cm^−1^, which guarantees the TDLAS system has a high resolution, SNR, and response capability. Firstly, such a TDLAS system was used to measure the temperature of the flat flame burner at different equivalence ratios and different flame heights. Compared with the results obtained by the CARS technique and theoretical calculation, the measurement errors of temperature were less than 4% and the variation trend of temperature was consistent. Finally, this TDLAS system was used for temperature testing on a scramjet model engine, which demonstrated a good dynamic range and fast response. In the future, this reported TDLAS system can be applied to multi-parameter testing of complex flow fields in scramjet engines, aero-engines, and other combustors.

## Figures and Tables

**Table 1:** Comparison of the main parameters of the absorption spectral lines (T = 1200 K).

*v* (cm^−1^)	*S* (cm^−2^atm^−1^)	*E”* (cm^−1^)	*γ_air_* (cm^−1^atm^−1^)	*γ_self_* (cm^−1^atm^−1^)
7153.749	1.849 × 10^−3^	2552.857	0.027	0.184
7154.354	4.868 × 10^−3^	1789.043	0.022	0.145

## Data Availability

The data presented in this study are available on request from the corresponding author.
